# Molting Alters the Microbiome, Immune Response, and Digestive Enzyme Activity in Mud Crab (*Scylla paramamosain*)

**DOI:** 10.1128/mSystems.00917-21

**Published:** 2021-10-12

**Authors:** Ming Zhang, Xinxu Zhang, Ngoc Tuan Tran, Zaiqiao Sun, Xusheng Zhang, Haihui Ye, Yueling Zhang, Hongyu Ma, Jude Juventus Aweya, Shengkang Li

**Affiliations:** a Guangdong Provincial Key Laboratory of Marine Biology, Institute of Marine Sciences, Shantou University, Shantou, China; b Shenzhen Key Laboratory of Marine Microbiome Engineering, Institute for Advanced Study, Shenzhen University, Shenzhen, China; c Fisheries College of Jimei University, Xiamen, China; Duke University School of Medicine

**Keywords:** molting, microbiota, mud crab, immune response, digestive system, immune system, molt

## Abstract

Molting is a crucial lifelong process in the growth, development, and reproduction of crustaceans. In mud crab (*Scylla paramamosain*), new exoskeleton, gills, and appendages are formed after a molting, which contributes to a 40 to 90% increase in body weight. However, little is currently known about the associations between molting and the dynamic changes of microbiota and physiological characteristics in mud crabs. In this study, the effects of molting on changes of the microbiome, immune response, and digestive enzyme activities in mud crabs were investigated. The results showed dynamic changes in the abundances and community compositions of crab-associated microbiota harboring the gills, subcuticular epidermis, hepatopancreas, midgut, and hemolymph during molting. Renewed microbiota was observed in the gills and midgut of crabs at the postmolt stages, which seems to be related to the formation of a new exoskeleton after the molting. A significant positive correlation between the expression of two antimicrobial peptide (AMP) genes (*Sp*ALF5 and *Sp*Crustin) and the relative abundance of two predominant microorganisms (*Halomonas* and *Shewanella*) in hemolymph was observed in the whole molt cycle, suggesting that AMPs play a role in modulating hemolymph microbiota. Furthermore, digestive enzymes might play a vital role in the changes of microbiota harboring the hepatopancreas and midgut, which provide suitable conditions for restoring and reconstructing host-microbiome homeostasis during molting. In conclusion, this study confirms that molting affects host-associated microbiota and further sheds light on the effects on the immune response and the digestive systems as well.

**IMPORTANCE** Molting is crucial for crustaceans. In mud crab, its exoskeleton is renewed periodically during molting, and this process is an ideal model to study the effects of host development on its microbiota. Here, multiple approaches were used to investigate the changes in microbial taxa, immune response, and digestive enzyme activity with respect to molting in mud crab. The results found that a renewed microbiota was generated in the gills and midgut of crab after a molt. A significant positive correlation between changes in the relative abundances of microbes (such as *Halomonas* and *Shewanella*) and the expression of AMP genes (*Sp*ALF5 and *Sp*Crustin) was observed in the hemolymph of crabs during the whole molt cycle, suggesting the modulation of hemolymph microbes by AMPs. Furthermore, the digestive enzymes were found to participate in the regulation of microbiota in hepatopancreas and midgut, consequently providing a suitable condition for the restoration and reconstruction of host-microbiome homeostasis during the molting. This study confirms that molting affects the microbial communities and concomitantly influences the immune and digestive systems in mud crabs. This is also the first time the homeostasis of the host and microbiome, and the associations between molting and physiological characteristics in crustaceans, have been revealed.

## INTRODUCTION

**FIG 1:**
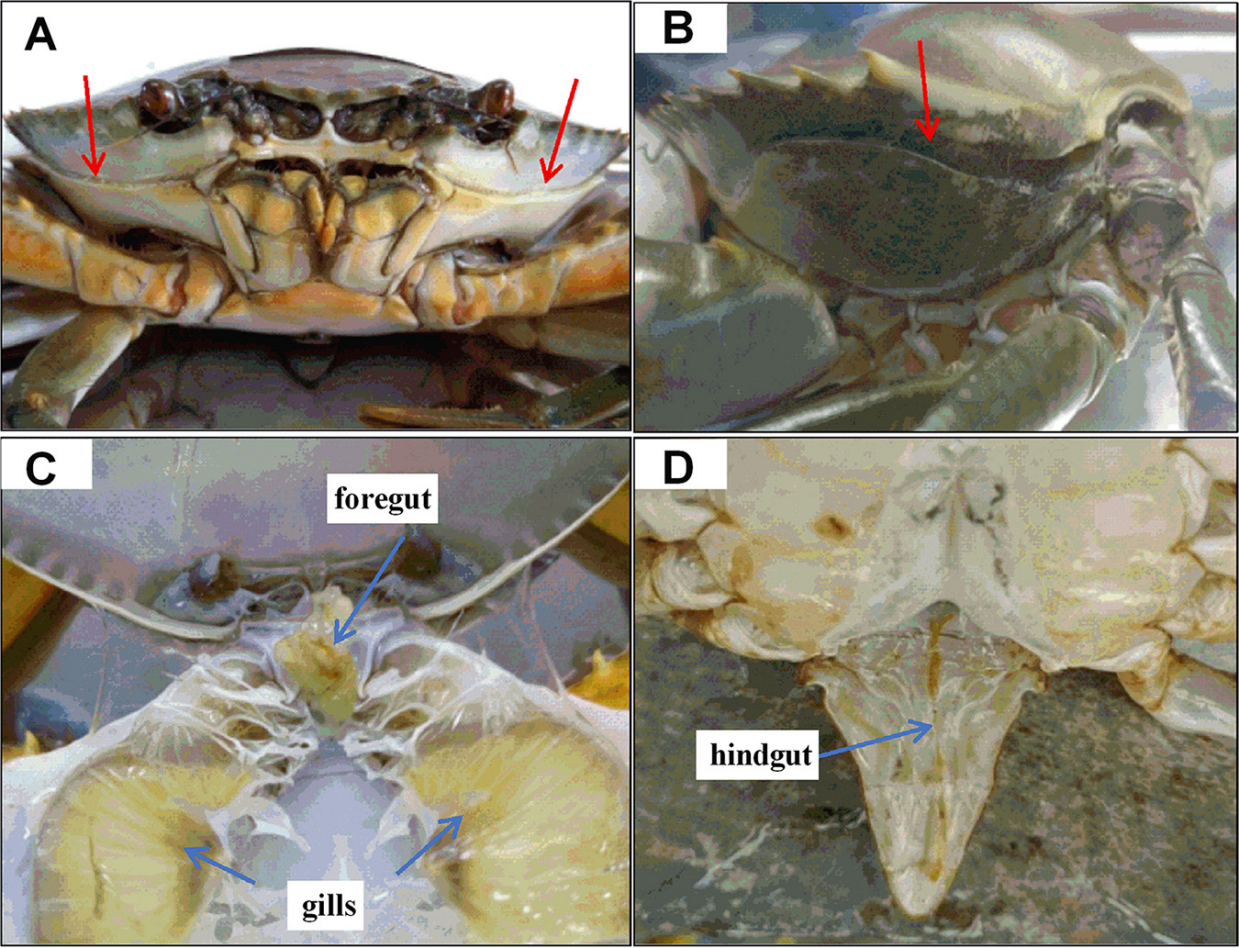
(A and B) Images of double-shelled crabs. The red arrows are cracks on the exoskeleton of the double-shelled crabs, indicating that they will molt within 1 to 3 days. (C and D) The old exoskeleton that is taken off the molted crab. The blue arrows indicate old tissues, including gill, foregut, outer membrane of the hindgut, and residual contents of the intestine.

Mud crab (*Scylla paramamosain*) is a commercially important aquaculture species on the coast of southeast China, with its annual production reported to be above 160,000 tons in 2019 (1). Molting is a crucial lifetime process for arthropods and some reptiles, which contributes to growth, development, reproduction, and appendage regeneration in animals (2, 3). In principle, both intrinsic factors (i.e., ecdysone, molt-inhibiting hormone, ecdysone receptor, and gonad development) (3, 4) and extrinsic factors (i.e., temperature, salinity, light, and nutrients) (5–7) control molting. In mud crab, molting occurs periodically to replace its old exoskeleton, including gills, foregut, the outer membrane of the hindgut, and residual contents of the intestine (Fig. 1) (8, 9). As a result, this process could be an ideal model to study host-microbiome interactions. In crabs, during molting, water and air are quickly absorbed into the body, simultaneously expanding the body size and weight until the new exoskeleton gradually hardens (10, 11). The increased weight gain, cleaner gills, and fewer or no gut contents are observed in the crabs at postmolting compared to premolting. However, the molting process substantially weakens crabs by consuming great energy, and the crabs often have to fast for 1 to 2 days after each molting (12).

Microorganisms are important inhabitants in vertebrates and invertebrates, and their community assembly is determined by several factors, such as the host’s phylogeny, body size, and lifestyle, as well as environmental conditions (13, 14). In fish and shellfish, previous studies have mainly focused on the effects of environmental factors (e.g., diets, culturing waters, and culture conditions) on changes in the composition of gut microbiota and the interactions between commensal microbes and their hosts (15–20). Hosts’ physiological development may also affect gut microbiota (20–25). Our previous studies have characterized the gut microbiota of healthy and diseased mud crabs and revealed an increase in the relative abundance of potential pathogens, such as *Vibrio*, *Aeromonas*, and *Hematodinium*, in the disease crabs compared with the healthy individuals (26, 27). Two *Bacillus* strains isolated from mud crab gut exhibit antibacterial activities and protect hosts against Vibrio parahaemolyticus infection (28). The predominant bacterial groups found in the hemolymph of mud crab are Escherichia*/Shigella* and *Halomonas* (29), and some of them originated from the digestive tract (30). Other studies have also shown decreases in the abundance of microbiota colonizing the hindgut of *Orthoporus ornatus* at the postmolting stage compared to premolting (31), and a decreased microbial diversity was found in the gut of newly molted *Amblyomma americanum* adults compared to the aged individuals (32). Similarly, recolonization by microorganisms in the branchial chamber of *Rimicaris exoculata* has been observed after molting (33). However, little is known about the dynamic changes and intrinsic factors of host-associated microbiota in an entire molt period of crustaceans, including mud crab.

This study explored the premise that molting affects crab microbiota and further studied the effect on the immune and digestive systems in mud crab. Five groups of mud crabs in this study were divided based on the key molt stages, i.e., double-shelled crabs (Ds) and 0, 3, 6, and 15 days postmolt. Changes in immunological parameters (such as hemocyte density, reactive oxygen species [ROS] level, antioxidant capacity, and gene expression) and activity of digestive enzymes (including lipase, chitinase, trypsin, and amylase) were correspondingly investigated in the hemolymph and hepatopancreas of mud crabs. The distinct patterns of the host’s physiological characteristics and microbiota were observed at pre- and postmolting.

## RESULTS

### Morphometric characteristics and digestive enzyme activity.

**FIG 2:**
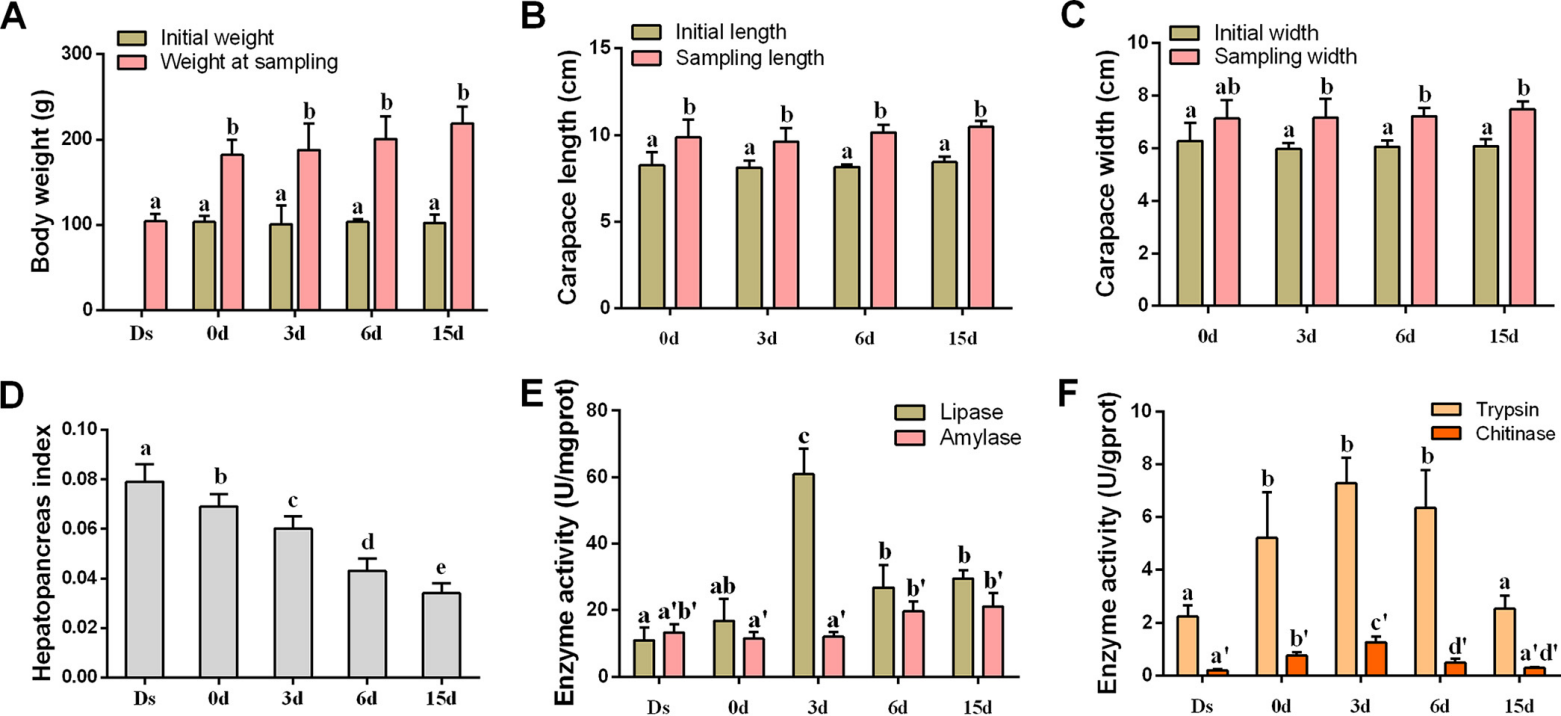
Changes of crab physiology and the activities of hepatopancreas digestive enzymes in a molt cycle. (A) Body weight. (B) Carapace length. (C) Carapace width. (D) Hepatopancreas index. (E) Lipase and amylase activity. (F) Trypsin and chitinase activity (gprot means "g protein"). Ds, double-shelled crab. The numbers 0d, 3d, 6d, and 15d represent the sampled crabs at different days after a molt. Significant differences (*P < *0.05) are represented by different letters.

Changes in the morphometric characteristics of crabs, including bodyweight, carapace length, and width, were measured at pre (Ds)- and postmolt (0 to 15 days) stages. The Ds crabs are those with a crack on their exoskeleton (Fig. 1) and molt within 1 to 2 days. The 0-day group indicates mud crabs finishing their molt within 8 h. The bodyweight of mud crabs at 0 days was significantly increased compared to those at Ds (with an average increase of 66.70%, *P < *0.05; Fig. 2A). The carapace length and width of crabs increased 20.16% and 18.86%, respectively, at 0 days compared with those at Ds (*P < *0.05; Fig. 2B and C). A gradual decrease in hepatopancreas index (HI) also occurred during the whole postmolt stages, with the highest and lowest values of 0.08 (Ds) and 0.03 (15 days), respectively (Fig. 2D).

The activity of digestive enzymes (i.e., lipase, chitinase, trypsin, and amylase) was higher in the hepatopancreas of crabs at 0 to 6 days than those at Ds (Fig. 2E and F). The highest activities of lipase (5.4-fold), chitinase (6.2-fold), and trypsin (2.6-fold) were observed at 3 days compared to Ds (*P < *0.05) (Fig. 2E and F). Amylase activity was increased at 6 days (48.91%) and 15 days (59.80%), while it was decreased at 3 days (−8.55%) compared to Ds (*P < *0.05) (Fig. 2E). However, the activities of lipase, chitinase, and trypsin at 15 days dropped to the same level as those at Ds (*P > *0.05) (Fig. 2E and F).

### Hemolymph conditions and expression of immune-related genes.

**FIG 3:**
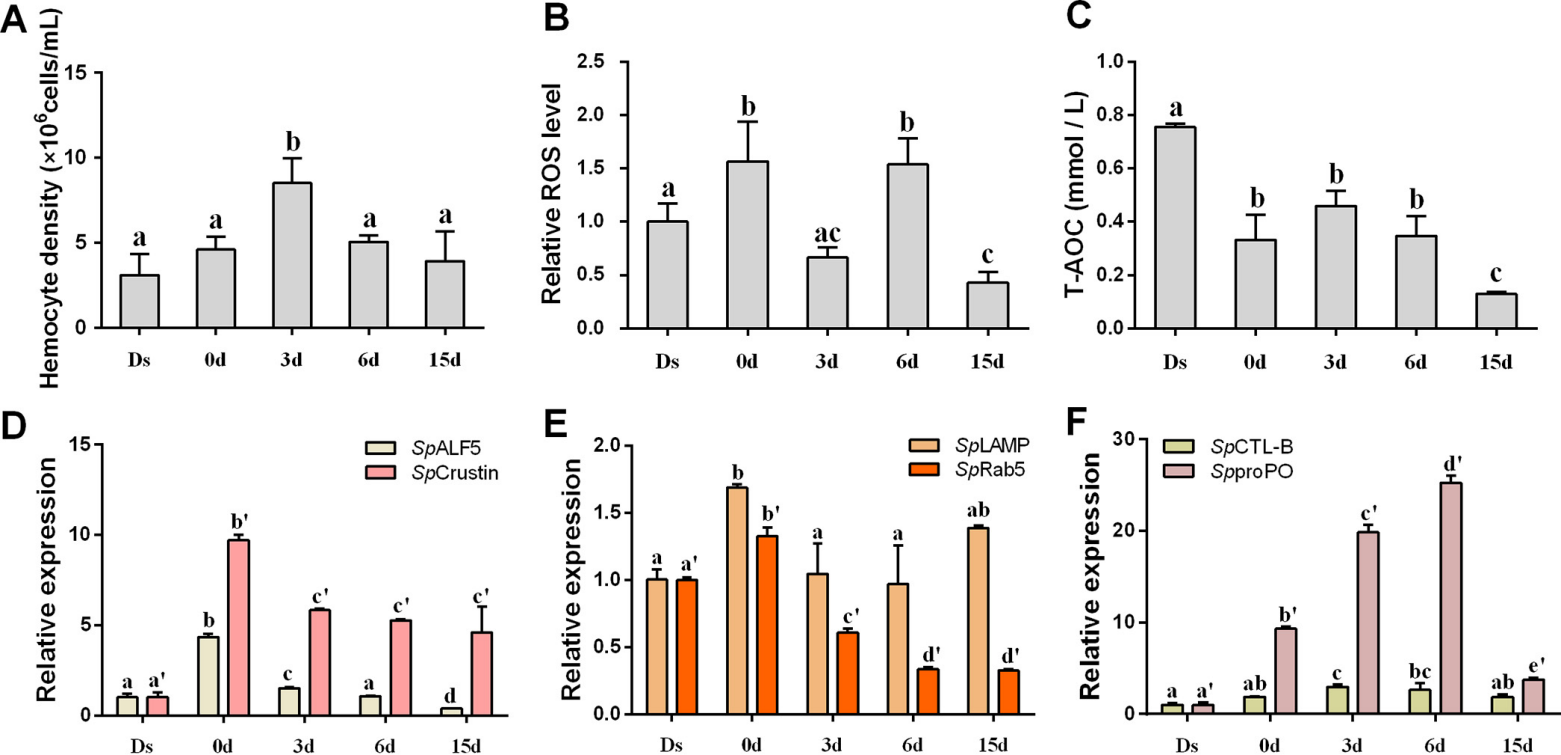
Hemolymph conditions and expression of immune-related genes in a molt cycle. (A) Hemocyte density. (B) Relative ROS level. (C) Antioxidant capacity. (D to F) Relative expression of immune-related genes. ROS, reactive oxygen species. T-AOC, total antioxidant capacity. Ds, double-shelled crab. The numbers 0d, 3d, 6d, and 15d represent the sampled crabs at different days after a molt. Significant differences (*P < *0.05) are represented by different letters.

The average number of hemocytes at the intermolt stage was 3.9 × 10^6^ cells/ml (15 days) (Fig. 3A). A significant increase in hemocyte density was observed only at 3 days (8.5 × 10^6^ cells/ml) (*P < *0.05; Fig. 3A). Compared to the Ds, the relative ROS level increased by 1.5-fold at 0 days and 6 days (*P < *0.05), and this value reduced to the basal level at 3 days and 15 days (Fig. 3B). The total antioxidant capacity (TAOC) in hemolymph showed a 56.00% decrease at 0 days compared to Ds (*P < *0.05), which remained at a low value of 0.13 mmol/liter at 15 days (Fig. 3C).

Transcript levels of two AMPs, *Sp*ALF5 and *Sp*Crustin, significantly increased by 3.3-fold and 8.5-fold at 0 days compared to Ds (Fig. 3D). The expression of *Sp*ALF5 was slightly low, whereas *Sp*Crustin was highly expressed in the hemolymph of crabs from 3 days to 15 days compared to Ds. The expression of two phagocytosis-related genes, *Sp*LAMP and *Sp*Rab5, was significantly increased at 0 days compared to Ds (*P < *0.05) and decreased to the basal level from 3 days to 15 days (Fig. 3E). The highest expression of *Sp*CTL-B and *Sp*proPO was found in crabs between 3 days and 6 days after molt (Fig. 3F).

### Changes in microbial abundance and composition during molting.

**FIG 4:**
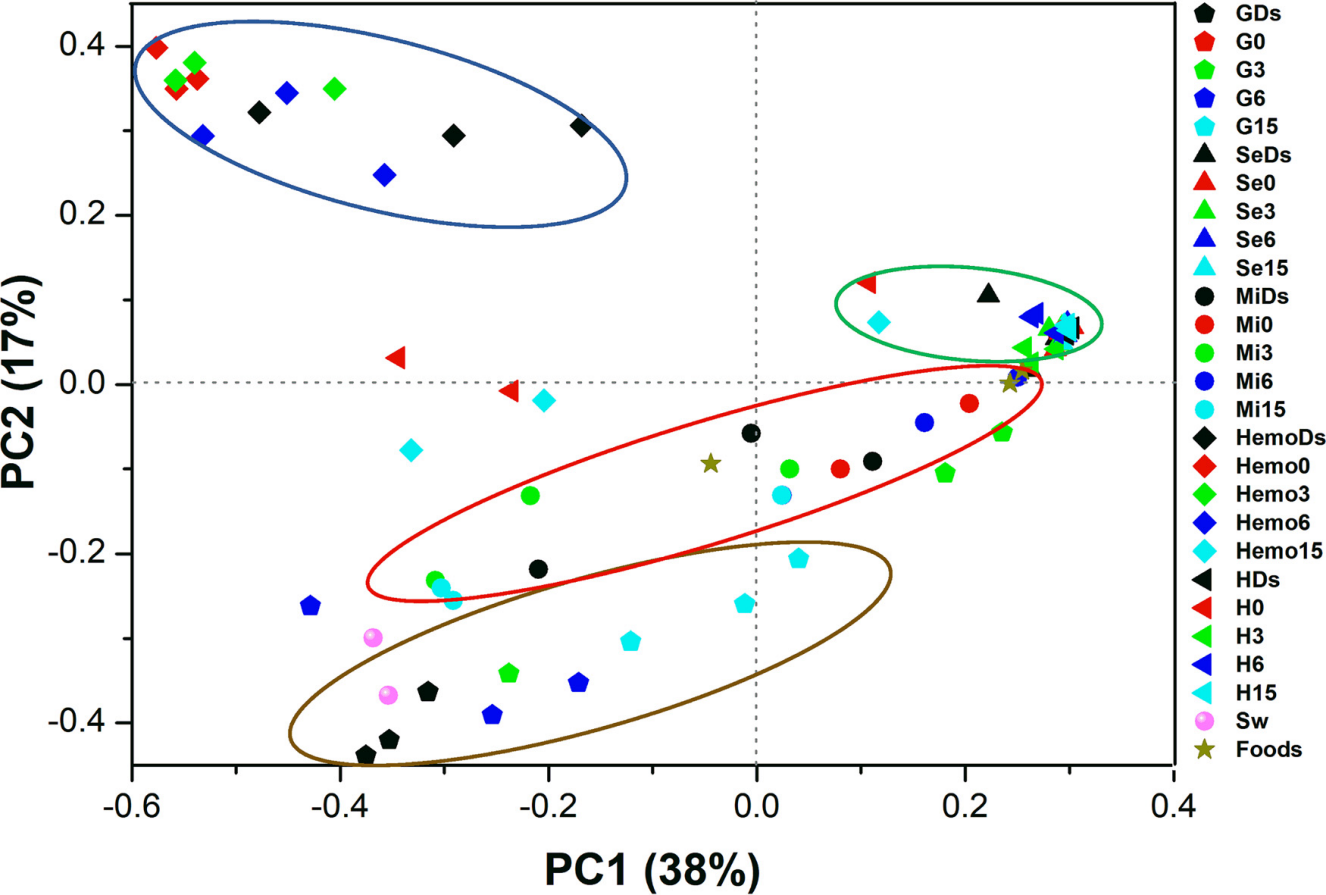
Principal coordinate analysis (PCoA) separates the crab microbiota with different tissues and molt stages. Different symbols and colors indicate different crab tissues and molt stages, respectively. G, gill; Se, subcuticular epidermis; Mi, midgut; Hemo, hemolymph; H, hepatopancreas; Sw, seawater; Ds, double-shelled crab. The numbers 0d, 3d, 6d, and 15d represent the sampled crabs at different days after a molt. The distances were determined using the Bray-Curtis method with relative abundances of microorganisms at the genus level. Three samples are sequenced for each group.

The highest microbial diversity was found in the culturing seawater and crab foods, as determined by the Shannon, Chao1, observed species, and PD whole tree indexes (see Fig. S1 in the supplemental material). Principal coordinate analysis (PCoA) revealed that the microbiota was tissue specific and stage related during the molting process (Fig. 4). The bacterial community structure in hemolymph was separated from those in other tissues (Fig. 4). However, the microbiotas between subcuticular epidermis and hepatopancreas were similar among different molt stages (Fig. 4). The shared microbial groups (at the genus level) in gills, subcuticular epidermis, midgut, hemolymph, and hepatopancreas were 62, 48, 32, 16, and 17, respectively (Fig. S2).

10.1128/mSystems.00917-21.1 FIG S1 Violin plots showing the distribution of alpha diversity indexes in different crab tissues, seawater, and foods. (A) Shannon, (B) Chao1, (C) observed species, (D) PD whole tree. G, gills. Se, subcuticular epidermis. Mi, midgut. Hemo, hemolymph. H, hepatopancreas. Sw, seawater. Ds, double-shelled crab. The numbers 0d, 3d, 6d, and 15d represent the sampled crabs at different days after a molt. The white dot indicates the median, and the box covers between the 25% and 75% quantiles. Download FIG S1, TIF file, 0.2 MB. Copyright © 2021 Zhang et al. 2021 Zhang et al. https://creativecommons.org/licenses/by/4.0/ This content is distributed under the terms of the Creative Commons Attribution 4.0 International license.

10.1128/mSystems.00917-21.2 FIG S2 Venn diagrams showing the shared bacterial groups in a specific crab tissue in a molt cycle (at genus level). G, gill. Se, subcuticular epidermis. Mi, midgut. Hemo, hemolymph. H, hepatopancreas. Ds, double-shelled crab. The numbers of 0, 3, 6, and 15 represent the sampled crabs at different days after a molt. Download FIG S2, TIF file, 0.2 MB. Copyright © 2021 Zhang et al. 2021 Zhang et al. https://creativecommons.org/licenses/by/4.0/ This content is distributed under the terms of the Creative Commons Attribution 4.0 International license.

**FIG 5:**
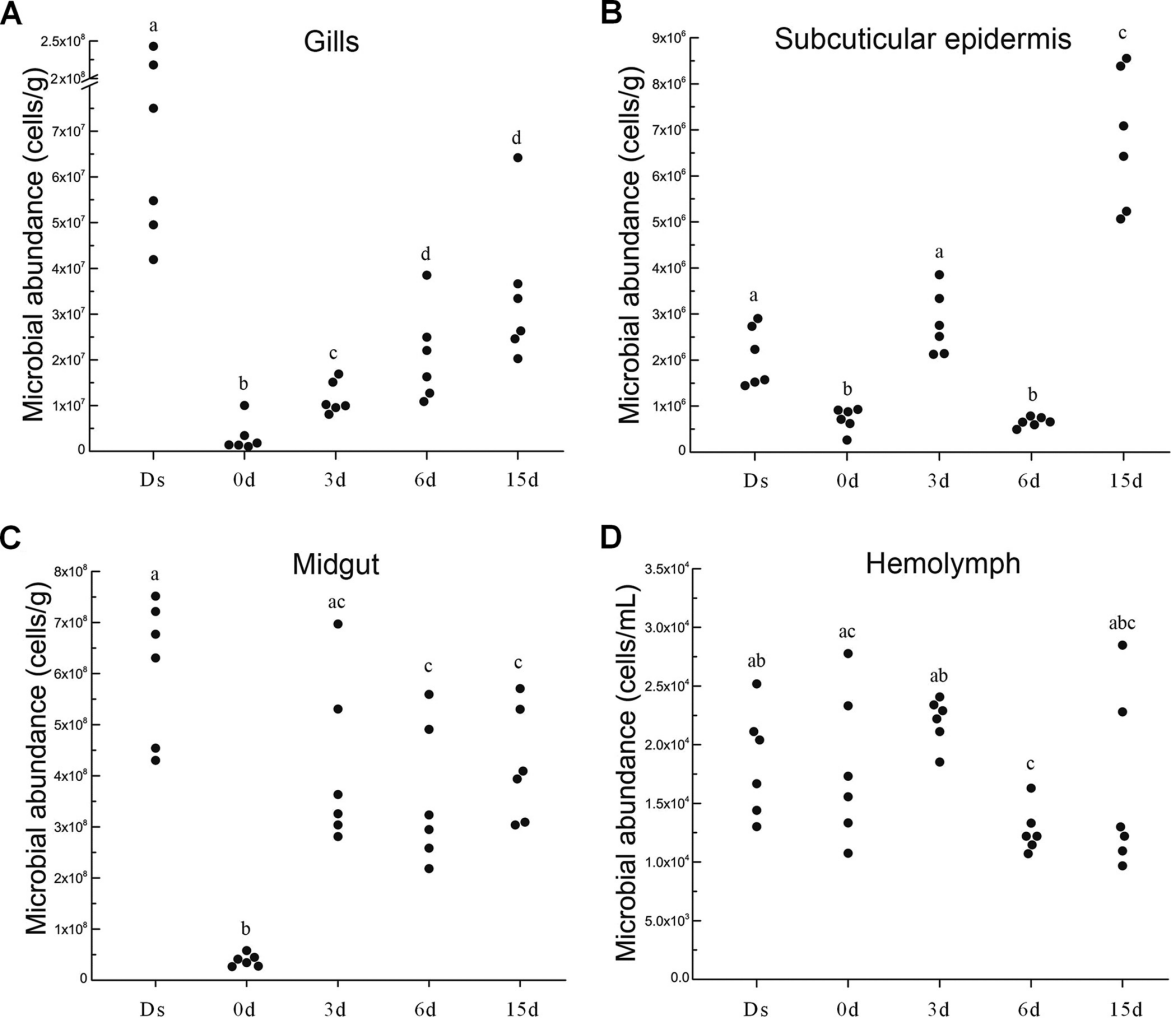
Microbial abundances in the gills (A), subcuticular epidermis (B), midgut (C), and hemolymph (D) of the crabs in a molt cycle. Each dot indicates an individual sample. Ds, double-shelled crab. The numbers 0d, 3d, 6d, and 15d represent the sampled crabs at different days after a molt. Significant differences (*P < *0.05) are represented by different letters.

**FIG 6:**
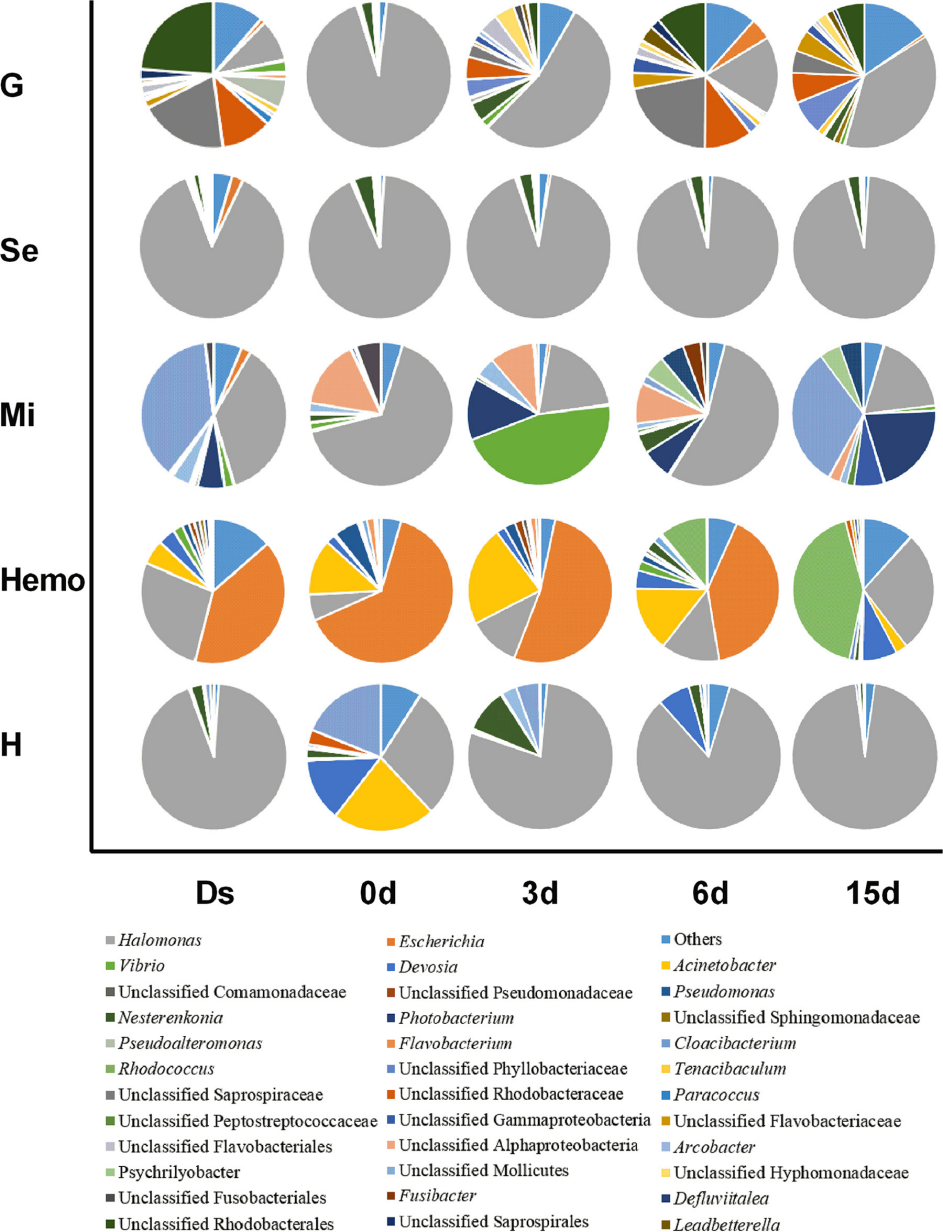
Microbial community compositions in different crab tissues in a molt cycle. G, gill; Se, subcuticular epidermis; Mi, midgut; Hemo, hemolymph; H, hepatopancreas. Ds, double-shelled crab. The numbers 0d, 3d, 6d, and 15d represent the sampled crabs at different days after molt. Detailed values from this figure are shown in Table S4.

In gills, the highest microbial abundance was in the crabs at Ds (1.1 × 10^8^ cells/g), and the lowest was at 0 days (3.1 × 10^6^ cells/g). There was a gradual increase from 0 days to 15 days, with the highest density of 3.4 × 10^7^ cells/g at 15 days (Fig. 5A). Based on the 16S rRNA gene sequencing results, the Venn diagram showed that a total of 36 bacterial genera disappeared after molt (Ds versus 0 days), while 41 genera were newly detected at 0 days compared to Ds crabs (Fig. S2A). The microbial diversity was lowest at 0 days, and the relative abundance of major bacteria also showed a distinct pattern compared to other molt stages. The relative abundances of unclassified *Rhodobacterales*, unclassified *Saprospiraceae*, *Vibrio*, and *Flavobacterium* were significantly decreased after molting (Ds versus 0 days) (*P* < 0.05), whereas the relative abundances of *Halomonas* and *Devosia* significantly increased from Ds to 0 days (*P* < 0.05) before decreasing from 3 days to 15 days (Fig. 6, Table S3A).

10.1128/mSystems.00917-21.9 TABLE S3 Metastat analysis of the relative abundances of microorganisms in gills (A), subcuticular epidermis (B), midgut (C), hemolymph (D), and hepatopancreas (E) at the genus level. Download Table S3, XLSX file, 0.02 MB. Copyright © 2021 Zhang et al. 2021 Zhang et al. https://creativecommons.org/licenses/by/4.0/ This content is distributed under the terms of the Creative Commons Attribution 4.0 International license.

In the subcuticular epidermis, the lowest abundances of microorganisms were at 0 days (7.2 × 10^5^ cells/g) and 6 days (6.5 × 10^5^ cells/g) (Fig. 5B). The highest microbial abundance (6.8 × 10^6^ cells/g) was found at 15 days, followed by 3 days (2.8 × 10^6^ cells/g) and Ds (2.1 × 10^6^ cells/g). The microbial diversity reached the highest level at Ds and maintained at the similar lower values between 0 days and 15 days (Fig. S1). In total, 135 bacterial groups disappeared at 0 days compared to Ds. Of these bacterial groups, 43, 13, and 7 genera were recovered at 3 days, 6 days, and 15 days, respectively (Fig. S2B). Twenty-five genera, including *Cetobacterium*, *Clostridium*, *Myroides*, *Lactococcus*, *Clostridium*, and *Lewinella*, were newly detected at 0 days (Fig. 6, Table S3B).

**FIG 7:**
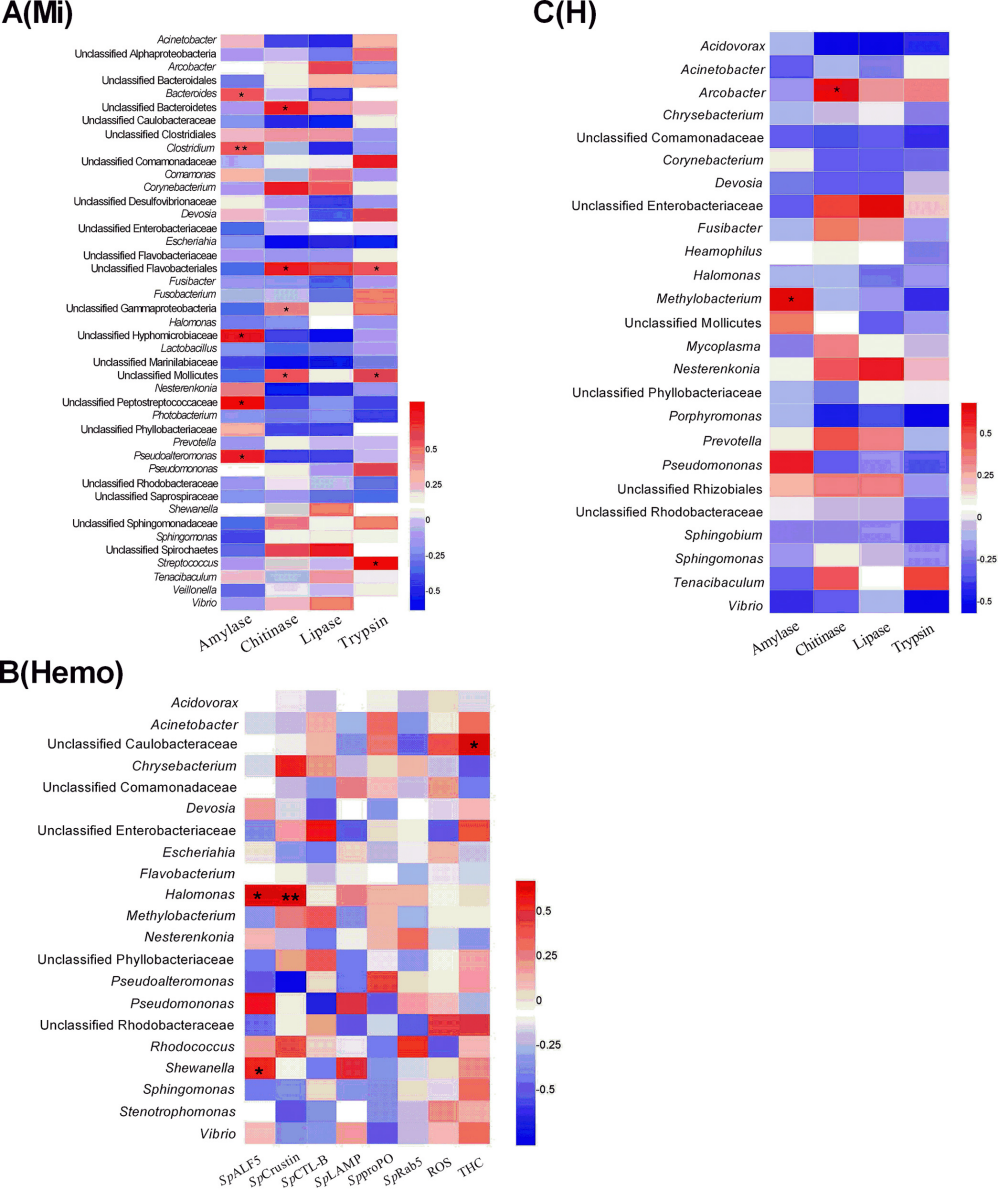
Mantel tests of the correlations among relative abundances of microbial groups, immune-related genes, and activities of digestive enzymes in a molt cycle. (A) Midgut (Mi). (B) Hemolymph (Hemo). (C) Hepatopancreas (H). The red or blue color represents a positive or negative correlation, respectively. The asterisks indicate that the correlation is statistically significant (*, *P < *0.05; **, *P < *0.01).

In the midgut, the microbial abundance was increased in the postmolt stages (0 days, 3.8 × 10^7^ cells/g; 3 days, 4.2 × 10^8^ cells/g; 6 days, 3.6 × 10^8^ cells/g; and 15 days, 4.2 × 10^8^ cells/g), although this was lower than that at Ds (6.1 × 10^8^ cells/g) (Fig. 5C). A total of five taxa showed distinct patterns in the whole molt period, including *Rhodobacteraceae* (at Ds), *Vibrionales* (at 3 days), and *Micrococcaceae*, *Actinomycetales*, and *Actinobacteria* (at 6 days) (Fig. S3C). The relative abundance of some bacterial groups, such as unclassified *Mollicutes* (Ds, 37.69%; 0 days, 0.20%; 15 days, 31.75%) and *Photobacterium* (Ds, 5.92%; 0 days, 0.12%; 15 days, 20.86%), were significantly decreased from Ds to 0 days and rapidly recovered at 15 days (*P < *0.05). Fifty-three bacterial taxa were newly identified at 0 days, but 40 of them (such as *Bradyrhizobium* and *Xanthobacter*) disappeared in the postmolt stages (Fig. 6, Table S3C). During the whole molt period, the changing trend of the relative abundances of *Bacteroides*, *Clostridium*, and *Pseudoalteromonas* were positively correlated with the amylase activity in the midgut (*P < *0.05; Fig. 7A).

10.1128/mSystems.00917-21.3 FIG S3 LEfSe identifies the most differentially abundant bacterial groups in a molt cycle, including (A) gill, (B) subcuticular epidermis, (C) midgut, (D) hemolymph, and (E) hepatopancreas. The linear discriminant analysis (LDA) scores are computed based on their relative abundances in each sample. G, gill. Se, subcuticular epidermis. Mi, midgut. Hemo, hemolymph. H, hepatopancreas. Ds, double-shelled crab. The numbers 0, 3, 6, and 15 represent the crabs on different days after a molt. Download FIG S3, TIF file, 0.2 MB. Copyright © 2021 Zhang et al. 2021 Zhang et al. https://creativecommons.org/licenses/by/4.0/ This content is distributed under the terms of the Creative Commons Attribution 4.0 International license.

In the hemolymph, the microbial abundances remained stable in a molt, with an average of 1.8 × 10^4^ cells/ml at Ds, 1.8 × 10^4^ cells/ml at 0 days, 2.2 × 10^4^ cells/ml at 3 days, 1.3 × 10^4^ cells/ml at 6 days, and 1.6 × 10^4^ cells/ml at 15 days (Fig. 5D). The relative abundance of *Halomonas* (Ds, 27.38%; 0 days, 5.86%; *P = *0.016), *Devosia* (Ds, 3.89%; 0 days, 2.05%; *P = *0.011), *Photobacterium* (Ds, 1.02%; 0 days, 0%; *P = *0.001), and *Shewanella* (Ds, 0.74%; 0 days, 0%; *P = *0.032) significantly decreased from Ds to 0 days. Some bacterial genera, such as *Paracoccus*, *Sphingobacterium*, *Serratia*, *Arcobacter*, *Deinococcus*, *Alteromonas*, and *Phaeobacter*, were detected throughout the molting cycle except for 3 days postmolt. Some bacterial taxa, such as unclassified *Phyllobacteriaceae*, Escherichia, Acinetobacter, Pseudomonas, *Flavobacterium*, *Delftia*, and *Marinomonas*, were significantly increased or newly detected after the molt (from Ds to 0 days) (Fig. 6, Table S3D). The relative abundances of *Halomonas* and *Shewanella* correlated positively with the hemocyte expression of the AMPs *Sp*ALF5 and *Sp*Crustin during molting, as revealed by the Mantel test (*P < *0.05; Fig. 7B).

In the hepatopancreas, 17 bacterial taxa (such as *Halomonas*, Acinetobacter, *Nesterenkonia*, and *Devosia*) changed their relative abundances throughout the molt cycle (Fig. 6). For example, the relative abundance of *Halomonas* was significantly changed from 93.24% (at Ds) to 28.86% (at 0 days) or 28.86% (at 0 days) to 78.71% (at 3 days) (*P < *0.05) (Fig. 6, Table S3E). The unclassified *Pseudomonadaceae* and *Devosia* showed a significant increase at 0 days compared with those at Ds (*P < *0.05) (Fig. 6, Table S3E). Seven taxa showed distinct patterns in the whole molt period, including *Sphingomonas* (at Ds), *Moraxellaceae* (at 0 days), *Halomonas* (at 0 days), *Devosia* (at 0 days), *Clostridiales* (at 3 days), unclassified *Caulobacteraceae* (at 6 days), and *Oceanospirillales* (at 15 days) (Fig. S3E). The relative abundances of *Arcobacter* and *Methylobacterium* correlated positively with the activity of hepatopancreas chitinase and amylase, respectively (*P < *0.05; Fig. 7C).

10.1128/mSystems.00917-21.10 TABLE S4 Microbial community compositions in different crab tissues in a molt cycle. Detailed values for Fig. 6. Download Table S4, XLSX file, 2.0 MB. Copyright © 2021 Zhang et al. 2021 Zhang et al. https://creativecommons.org/licenses/by/4.0/ This content is distributed under the terms of the Creative Commons Attribution 4.0 International license.

## DISCUSSION

This study shows that various tissues of mud crabs harbored distinct relative abundance and communities of microorganisms, which confirms the hypothesis that host-associated microorganisms are tissue specific (34). Because the gills, subcuticular epidermis, and gut of mud crabs are in direct contact with the surrounding seawater, these organs may accumulate beneficial and harmful substances (including microorganisms) from the environment (19, 35). The symbiotic microorganisms harboring hosts’ bodies seem to change their diversity and community composition at different developmental stages, such as molting and metamorphosis (20, 22, 23). In this study, great differences in mud crab’s morphometric characteristics (body weight, carapace length, width, and HI), the activity of digestive enzymes, immune response (the expression of immune-related genes), and colonized microbiota (abundance and community composition) were observed at 0 days postmolt compared to those at premolt (Ds) and 15 days postmolt.

The microbial density in the gills dramatically decreased to the lowest level at 0 days, which gradually recovered during the postmolt period. Notably, the relative abundance of some potential pathogens, such as *Flavobacterium*, *Pseudoalteromonas*, and *Vibrio*, decreased after molting, suggesting that molting plays an essential role in removing pathogenic bacteria from the gills of mud crabs. In mud crab, gills are responsible for gaseous exchange, osmolyte transport, nitrogenous excretion, acid-base balance, volume regulation between the body and external environment, and protection against infection and also act as a potential hematopoietic organ (36–39). The highest microbial cell density was found in the gills of crabs at the premolt stage, which might be related to the direct contact with the surrounding environment of gills during respiration, water absorption, and microbial filtration.

**FIG 8:**
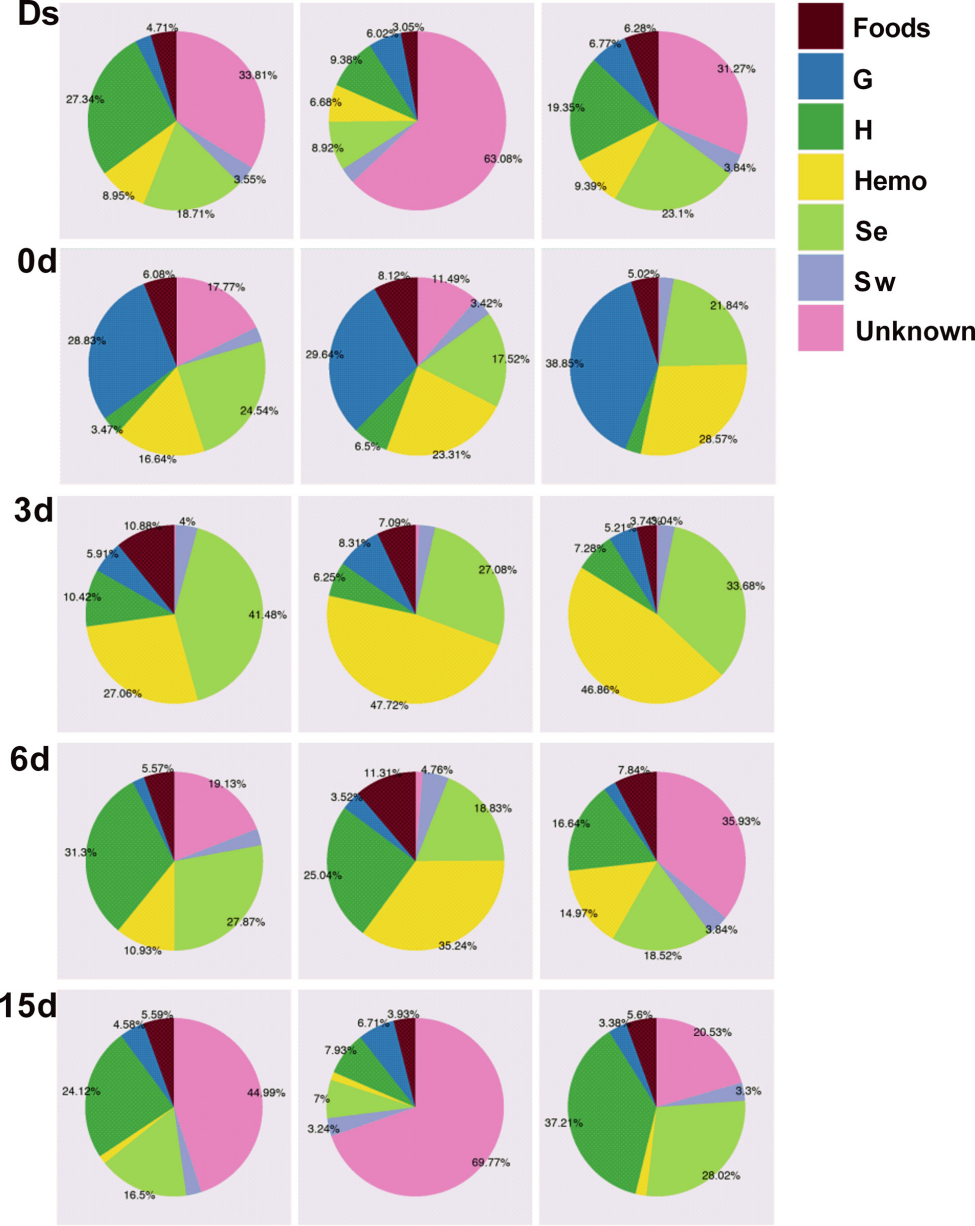
SourceTracker diagrams of the microbiota in the midgut of mud crabs (at phylum level). G, gill. Se, subcuticular epidermis. Hemo, hemolymph. H, hepatopancreas. Sw, seawater. Foods, crab foods, which are a mixture of three animals, including *Ruditapes philippinarum*, *Cipangopaludina cahayensis*, and *Sinonovacula constricta*. Ds, double-shelled crab. The numbers 0d, 3d, 6d, and 15d represent the sampled crabs at different days after a molt. Three samples are sequenced for each group.

Microbial numbers decreased to the lowest in midgut at 0 days (3.8 × 10^7^ cells/g) but recovered at 3 days (4.2 × 10^8^ cells/g) to reach levels that are similar to those at 15 days (4.2 × 10^8^ cells/g) and Ds (6.1 × 10^8^ cells/g), which could be attributed to the observation that crabs cease feeding during molting (40). At a period of 3 to 15 days postmolt, an increase in the relative abundance of microbiota (e.g., *Photobacterium*, Escherichia, *Vibrio*, and *Arcobacter*) in the midgut was observed, which might come from the surrounding seawater and feed (22, 41). Interestingly, SourceTracker analysis showed that some microorganisms in the midgut were from other tissues, such as gills, hemolymph, hepatopancreas, and subcuticular epidermis (Fig. 8). Taken together, microorganisms originated from the surrounding environment and other tissues are important sources of gut microbiota in mud crabs, as previously reported (15, 16, 20, 29).

However, the core microbiota in these mud crab tissues did not change during the whole molt cycle, although some microorganisms are separated from the old tissues. The translocation and reconstruction of the core microbiota in different tissues cooccur with the molting process. These events are also regulated by the physiological response of the host (through the expression of immune-related genes and modulation of digestive enzyme activity) and environmental factors (foods and seawater), which finally maintain the homeostasis between crabs and their associated microbiota (42).

The results of this study revealed an upregulation in the expression of genes involved in antimicrobial response (*Sp*ALF5 and *Sp*Crustin), phagocytosis (*Sp*LAMP and *Sp*Rab5), agglutination (*Sp*CTL-B), and melanization (*Sp*proPO) in the hemolymph of mud crabs at 0 days compared with those at Ds, which indicates the role of molting in activating host immune response. Antimicrobial peptides (AMPs) are essential immune molecules that protect hosts against exogenous microorganisms. Hemocytes of healthy Pacific oysters could express basal levels of two AMPs (defensins and proline-rich peptides), which indicates that hemolymph microorganisms stimulate the generation of these AMPs to boost host immunity (43). Moreover, the results obtained previously showed the high expression of *Sp*ALF5 and *Sp*Crustin genes in the hemolymph of mud crab challenged with Vibrio parahaemolyticus (34, 44, 45). In the current study, the expression of AMPs (*Sp*ALF5 and *Sp*Crustin) correlated positively and significantly with the relative abundances of *Halomonas* and *Shewanella* during molting (Fig. 7A), which indicates the role of AMPs in modulating the abundance of these two bacterial genera in mud crab hemolymph. As previously reported, AMPs can induce the transcription of immune effectors that protect the host against bacterial invasion through IMD and Toll signaling pathways (46, 47) and their downstream molecules (i.e., Dorsal, Relish, and Caudal) to maintain homeostasis. However, *Halomonas* and *Shewanella* genera are opportunistic pathogens (29, 48–50). For example, exposure to Microcystis aeruginosa increased the relative abundance of pathogenic microorganisms such as *Halomonas* and *Shewanella* in the zebrafish intestine, which enhanced inflammatory response and produced detrimental effects (51). *Shewanella* spp. are occasionally isolated from diseased aquatic animals such as fish and shrimps and may facilitate the increase in the hemolytic activity of V. parahaemolyticus in a time-dose-temperature-dependent manner (52, 53). Furthermore, hemocyte agglutination and phagocytosis are important in immune defense against pathogens, with increased ROS levels involved in eliminating bacteria pathogens in crabs and shrimps (54, 55). At 0 days after molting, the significant increase in *Sp*CTL-B (a C-type lectin gene), two phagocytosis-related genes, *Sp*LAMP and *Sp*Rab5, coupled with the increased ROS level, indicates an immune response by the crabs to invasion by the exogenous bacteria or proliferation of indigenous bacteria. This observation is probably explained by the important role of hemolymph in the host immune response. The microbiota colonizing the hemolymph are tightly controlled by host-microbiome homeostasis immunity (29), which is confirmed here by the stability of microbial abundances during a molt cycle in the hemolymph of mud crabs. A positive correlation between total hemocyte counts (THC) and the relative abundance of unclassified *Caulobacteraceae* was observed in present study. *Caulobacteraceae* are a family of nonfilamentous bacteria that form stalks or zoogloeal masses of ferric hydroxide (56), which indicates that the *Caulobacteraceae* found in hemolymph live a syntrophic or epibiotic lifestyle by colonizing or attaching themselves to the surface of hemocytes, as reported previously (29).

In the digestive system, the interactions between digestive enzymes and the abundance of microorganisms are versatile. Digestive enzymes are produced and secreted by the host to digest fats, proteins, and carbohydrates in foods to provide nutrients for the growth and development of the host (57). Gut microorganisms can further use digestible and/or indigestible debris (e.g., dietary fibers) as carbon and energy sources (19, 58). In the present study, the highest activity of digestive enzymes was found 3 days postmolt, which possibly relates to the feeding habit of mud crabs during a molt cycle. Molting is an extremely energy-consuming process that lasts 1 to 2 days, with crabs staying still in their shelters and not feeding during and immediately after molting (40). Crabs might only start to eat at 3 days postmolt; thus, the higher digestive enzyme activity facilitates more efficient assimilation of nutrients. Interestingly, in fish and other vertebrates, gut microbiota (such as *Clostridium*, *Actinomyces*, and *Ruminococcus*) are capable of synthesizing digestive enzymes (such as cellulase, xylanase, pectinase, and/or β-glucanase) (59–62). Here, changes in the relative abundance of microbiota in the hepatopancreas and midgut of mud crabs are correlated with digestive enzyme activities during the whole molt period. For example, in the hepatopancreas, the relative abundances of *Arcobacter* and *Methylobacterium* were significantly related to the activity of chitinase and amylase (Fig. 7B). The results agree with the previous findings that *Arcobacter* spp. can produce chitinase and lysozyme and participate in the biodegradation of shrimp shells (63). *Methylobacterium* spp. can produce cellulase, pectinase, lipase, amylase, and chitinase (64). The bacterial genera *Bacteroides*, *Clostridium*, and *Pseudoalteromonas* are significantly correlated with amylase activity in the midgut of mud crabs during molting (Fig. 7C), which is consistent with the results of previous studies that many members of these genera can produce amylase (65–67). Collectively, the results of this study suggest that digestive enzymes could regulate the microbiota in the hepatopancreas and midgut of mud crab during molting, which provides a suitable condition for the restoration and reconstruction of host-microbiome homeostasis in crab molting.

In conclusion, this study confirms the hypothesis that molting affects the microbiota in the tissues of mud crabs. Renewed microbiota was generated in gills and midgut after a molt, and dynamic changes of crab-associated microbiota are observed in a molt cycle. This study suggests that the molting would further alter the immune response and the digestive systems in mud crab as well. Future studies using metagenomics, metatranscriptomics, and/or culturomics are needed to identify the potential functions of the host-associated microorganisms during molting in mud crabs.

## MATERIALS AND METHODS

### Ethics statement.

The mud crabs used in this study were obtained from a local aquaculture farm in Xiamen, Fujian, China. The animals were processed according to the Regulations for the Administration of Affairs Concerning Experimental Animals established by Guangdong Provincial Department of Science and Technology on animal use and care. The experiments were approved by the Institutional Animal Care and Use Committee of Shantou University.

### Mud crab aquaculture and environmental parameters of the seawater.

In general, male crabs (100 ± 20 g), which were healthy with no signs of disease, had complete appendages, and no material attachments on their body surfaces, were used. Before the experiments, crabs were cultured in a “crab house” aquaculture system equipped with a continuous recirculating seawater system. The seawater used was drawn from nearby natural seawater, which was then passed through a filter system containing activated carbon and silica sand, with the seawater’s daily exchange capacity maintained at 600 to 800% in volume. Crabs were individually kept in the units of the crab house (40 by 25 by 18 cm) to avoid fighting or being killed during molting. The crab house was cleaned once daily to remove crab feces and residual feeds. Crabs were fed daily with a mixture of *Ruditapes philippinarum*, *Cipangopaludina cahayensis*, and *Sinonovacula constricta* at a total amount of 10 to ∼20 g per day. During the culturing period, the average temperature (24.6°C), salinity (20.9 ‰), pH (7.9), and nitrate (35.4 mg/liter) and ammonium concentrations (0.05 mg/liter) in the seawater were relatively stable (see Fig. S4 and S5 in the supplemental material), and the dissolved oxygen concentration was maintained at 8.15 ± 0.36 mg/liter every day. Molting was observed and recorded three times daily (at 8:00, 16:00, and 24:00, respectively). Most of the crabs were molted at two time intervals, including October 19 to 31 and November 7 to 11 in 2016 (Fig. S4). In this study, five experimental groups were set up, and six crabs were used in each group. The five groups included premolt crabs (double-shelled crabs, or Ds), 0 days postmolt, 3 days postmolt, 6 days postmolt, and 15 days postmolt.

10.1128/mSystems.00917-21.4 FIG S4 Crab molting and environmental conditions during the sampling period. The black vertical bar at the bottom indicates the number of molted crabs at a certain date. The orange bar indicates the sampling date of the double-shelled crabs. The temperature is measured once every hour. The salinity is measured once every day, and two sudden drops of the seawater salinity were due to rainfall events (October 10 and October 24). Average T., Max T., and Min T. are the average, highest, and lowest temperatures in a day. Download FIG S4, TIF file, 2.1 MB. Copyright © 2021 Zhang et al. 2021 Zhang et al. https://creativecommons.org/licenses/by/4.0/ This content is distributed under the terms of the Creative Commons Attribution 4.0 International license.

10.1128/mSystems.00917-21.5 FIG S5 Concentrations of nitrate, ammonium, and pH of the culturing seawater during the sampling period. Three replicate samples are measured for each point. Download FIG S5, TIF file, 2.2 MB. Copyright © 2021 Zhang et al. 2021 Zhang et al. https://creativecommons.org/licenses/by/4.0/ This content is distributed under the terms of the Creative Commons Attribution 4.0 International license.

### Morphometric characteristics.

The body weight, carapace length, and width of each crab were measured at the beginning (when the crabs were introduced to the crab house) and end (the time of sample collection) of each experiment. The hepatopancreas index (HI) was calculated according to the formula hepatopancreas index (hi) = total hepatopancreas weight/bodyweight.

### Sample collection and microbial cell enumeration.

The sampling procedure was performed in a sterile laminar flow hood, with the surface of each crab disinfected and wiped three times with 75% (vol/vol) ethanol. Hemolymph was withdrawn via the arthropodal membrane (at the bases of the walking legs) using a 1-ml disposable syringe into a tube containing precooled sterile anticoagulant buffer (450 mM NaCl, 100 mM glucose, 26 mM citric acid, 30 mM sodium citrate, pH 4.6) at a ratio of 1:1. Two hundred microliters of the anticoagulant-hemolymph mixture was fixed with 4 ml of sterile formalin solution (20 g/liter NaCl, 30 ml/liter formalin) for 2 h and stored at 4°C for cell enumeration (three replicates). The gills, subcuticular epidermis (a thin tissue that attached to the inner part of the shell), and midgut were collected into 2-ml sterile tubes with separate flame-sterilized scissors, which were then cut into pieces (<0.2 cm^2^). Pieces of food (including *R. philippinarum*, *C. cahayensis*, and *S. constricta*) used for feeding the mud crabs were collected and cut into small pieces (<0.2 cm^2^). Tissue samples were quickly weighed, and 0.1 g of each sample was preserved in 500 μl of 1× phosphate-buffered saline (PBS)-ethanol (vol/vol, 1:1) before being stored at −20°C until further analyses. One liter of culturing seawater was filtered through a sterile filter with a 0.22-μm mesh membrane (GPWP04700; Millipore, USA) using a vacuum pump, and the microbial cells from the culturing seawater were collected by direct filtering of the water. Half of the membranes were stored at −20°C for DNA extraction, and the other half were fixed with sterile formalin solution for 2 h and stored at 4°C for cell counting.

The anticoagulant-hemolymph-formalin mixture was processed as described previously (29). For gills, subcuticular epidermis, midgut, and crab food, the preserved samples were thawed and vortexed for 30 min (30). The mixture was diluted to the appropriate volume with sterile formalin solution (20 g/liter NaCl, 30 ml/liter formalin) and fixed for 2 h. The microbial mixture was captured by a 0.2-μm mesh membrane (GTBP02500; Millipore, USA).

All the collected samples containing microbial cells were stained with SYBR green I solution (1:40 vol/vol SYBR green I in 1× Tris-EDTA buffer) for 20 min. After staining, the dye was removed and the membrane was immersed in 25 μl of 10% glycerin. Cells were counted at ×1,000 magnification using a fluorescence microscope (ECLIPSE 90i; Nikon, Japan) with a blue filter as described elsewhere (68, 69). Each sample was extracted and counted in triplicates.

### DNA extraction, PCR amplification, and sequencing.

The total microbial DNA of hemolymph, gills, subcuticular epidermis, midgut, hepatopancreas, and crab foods were extracted using the PowerFecal DNA isolation kit (MO BIO Laboratories, USA) according to the manufacturer’s protocols. The hypervariable V3-V4 regions of the bacterial 16S rRNA genes were amplified by PCR using primers 341F and 806R (Table S1). PCRs were performed in triplicates with a 20-μl mixture containing 4 μl of 5× FastPfu buffer, 2 μl of 2.5 mM deoxynucleoside triphosphates, 5 μM forward and reverse primers, 0.4 μl of FastPfu DNA polymerase (TransStart, China), and 10 ng of template DNA and made up to the required volume with sterile water. The PCR conditions were 95°C for 5 min, 27 cycles at 95°C for 30 s, 55°C for 30 s, and 72°C for 45 s, and a final extension at 72°C for 5 min. The PCR products were then extracted from the 1% agarose gels and purified with a DNA gel extraction kit (Axygen, Hangzhou, China) according to the manufacturer's instructions. The concentration of the purified DNA was quantified using NanoDrop 2000 spectrophotometer. Equal molar amounts of each purified PCR product were pooled and sequenced on an Illumina HiSeq 2500 platform (2 × 250 bp) according to standard protocols.

10.1128/mSystems.00917-21.7 TABLE S1 PCR primers used in this study. Download Table S1, XLSX file, 0.01 MB. Copyright © 2021 Zhang et al. 2021 Zhang et al. https://creativecommons.org/licenses/by/4.0/ This content is distributed under the terms of the Creative Commons Attribution 4.0 International license.

### Determination of total hemocyte counts, reactive oxygen species, and total antioxidant capacity in hemolymph.

Approximately 20 μl of hemolymph was placed in a hemocytometer for total hemocyte count using an inverted phase-contrast microscope (Carl Zeiss Tessar Axio Lab, Germany). All hemocytes in both the top and bottom fields (1 by 1 mm) of the hemocytometer were counted. One milliliter of diluted hemolymph was used to measure ROS levels with a reactive oxygen species assay kit (Beyotime Biotechnology, China). Briefly, hemolymph was centrifuged at 800 × *g* for 20 min at 4°C to isolate hemocytes, followed by washing with 1× PBS and resuspended with 200 μl of 1× PBS containing 10 μM DCFH-DA. The mixture was incubated (protected from light) for 20 min at 37°C, after which the fluorescence intensity was measured at 488 and 525 nm using an Infinite 200 PRO NanoQuant microplate reader (Tecan, Switzerland). Total antioxidant capacity was determined using a commercial test kit (number A015-2-1) according to the manufacturer's instructions (Nanjing Jiancheng Bioengineering Institute, China). The optical density was measured at 405 nm using a microplate reader.

### Determination of hepatopancreas digestive enzyme activity.

The hepatopancreas of each crab was weighed and homogenized with 1× PBS before being centrifuged at 3,000 × *g* for 10 min at 4°C. The supernatant was used to measure enzymatic activities of lipase, amylase, and trypsin using commercial test kits (numbers A054-2-1, C016-1-1, and A080-2-2, respectively) (Nanjing Jiancheng Bioengineering Institute, China) according to the manufacturer's instructions. An increase of 0.001 per min is defined as one active unit (U). Chitinase activity was measured using an enzyme-linked immunosorbent assay kit (CK-EN95851; Shanghai Guchen Biotechnology, China) according to the manufacturer's instructions.

### RNA extraction and RT-qPCR.

The expression of six immune-related genes representing the four major types of innate immunity in mud crab were measured, including those of antimicrobial response (*Sp*ALF5 and *Sp*Crustin), phagocytosis (*Sp*LAMP and *Sp*Rab5), agglutination (*Sp*CTL-B), and melanization (*Sp*proPO) (44, 70–74). The hemocytes of crabs at various molt stages were collected by centrifuging hemolymph at 800 × *g* for 20 min at 4°C. Total RNA from hemocytes was extracted using TRIzol reagent (Ambion, USA). First-strand cDNAs were synthesized using the PrimeScript RT reagent kit with gDNA Eraser (TaKaRa, Dalian, China) according to the manufacturer’s instructions. The quantitative real-time PCR (RT-qPCR) analysis used the SYBR Premix Ex Taq II kit (Perfect Real Time) (TaKaRa, Dalian, China) by following the manufacturer’s instructions on a LightCycler 480 (Roche, USA). The RT-qPCR total reaction volume was 20 μl containing 10 μl of SYBR Premix Ex Taq II, 2 μl of the 4-fold diluted cDNA, 0.8 μl (10 μM) each of the forward and reverse primers, and 6.4 μl of ultrapure water. The primers used for the six immune-related genes (including *Sp*ALF5, *Sp*Crustin, *Sp*LAMP, *Sp*Rab5, *Sp*CTL-B, and *Sp*proPO) are in Table S1. The amplification procedure included a denaturation step of 95°C for 30 s, followed by 40 cycles of 95°C for 5 s, 60°C for 20 s, and then a melting curve analysis from 65°C to 95°C (74, 75). Triplicate samples were used for all experiments. The relative transcript level of each gene was determined with the β-actin gene as an internal control (75).

### Microbial community composition and SourceTracker analysis based on the 16S rRNA genes.

The raw sequence data analysis used a previously described procedure (29, 30). After removing the chimeric sequences, 39,750 effective sequences, on average, were randomly sampled for each sample to maintain the same sequencing depth (Table S2). The sequences were clustered into operational taxonomic units (OTUs) at a 97% sequence similarity cutoff using the Uparse package (version 7.0.1001) (76), and all OTUs were assigned to the Greengenes database by the QIIME software pipeline. For the SourceTracker analysis, the OTU table derived from quality filtering and OTU picking of the 16S rRNA gene sequences was used as the input file. This tool compares the community profiles in the “source” groups (i.e., gill, hepatopancreas, hemolymph, subcuticular epidermis, culturing seawater, and crab foods) with those in the “sink” group (i.e., midgut) by using Bayesian methods to calculate the contribution of each source to the sink. The SourceTracker analysis was performed using the default parameters: rarefaction depth, 1,000; burn-in, 100; restart, 10; and alpha (0.001) and beta (0.01) Dirichlet hyperparameters.

10.1128/mSystems.00917-21.8 TABLE S2 Number of bacterial 16S rRNA gene sequences used in this study. Download Table S2, XLSX file, 0.01 MB. Copyright © 2021 Zhang et al. 2021 Zhang et al. https://creativecommons.org/licenses/by/4.0/ This content is distributed under the terms of the Creative Commons Attribution 4.0 International license.

### Statistical analysis.

The statistical significance between two samples of crab physiology, hepatopancreas digestive enzymes, hemolymph conditions, expression of immune-related genes, and microbial abundances was analyzed using the Mann-Whitney U test (SPSS 22.0). Metastat analysis with Fisher’s exact test was performed in R software (version 3.2.0) (77) to determine the differences in the relative abundances of the microbiota of crab at different molt stages. The PCoA plot was performed in R using Bray-Curtis distances with the relative abundance of microorganisms at the genus level. Alpha diversity indices (i.e., Shannon, Chao1, observed species, and PD whole tree) were calculated by alpha_diversity.py in QIIME. The Mantel test was performed in R with the relative abundance of microorganisms at the genus level and measured immune-related genes/digestive enzymes at different molt stages. The differences were considered statistically significant at a *P* value of <0.05. The LDA effect size (LEfSe) analysis was performed with an LDA score threshold of >2.0.

### Data availability.

The 16S rRNA gene sequences of this study were deposited in the Sequence Read Archive of the National Center for Biotechnology Information (NCBI) under the accession number PRJNA640924.

10.1128/mSystems.00917-21.6 FIG S6 Microbial community compositions of the culturing seawater (A) and crab foods (B). The crab foods are the mixture of three animals, including *R. philippinarum*, *C. cahayensis*, and *S. constricta*. Download FIG S6, TIF file, 0.5 MB. Copyright © 2021 Zhang et al. 2021 Zhang et al. https://creativecommons.org/licenses/by/4.0/ This content is distributed under the terms of the Creative Commons Attribution 4.0 International license.
