# Supplementary figures and images for: Molting Alters the Microbiome, Immune Response, and Digestive Enzyme Activity in Mud Crab (Scylla paramamosain)
Source: mSystems. 2021 Oct 12;6(5):e00917-21. doi: 10.1128/mSystems.00917-21 (PMC8510556; doi:10.1128/mSystems.00917-21)

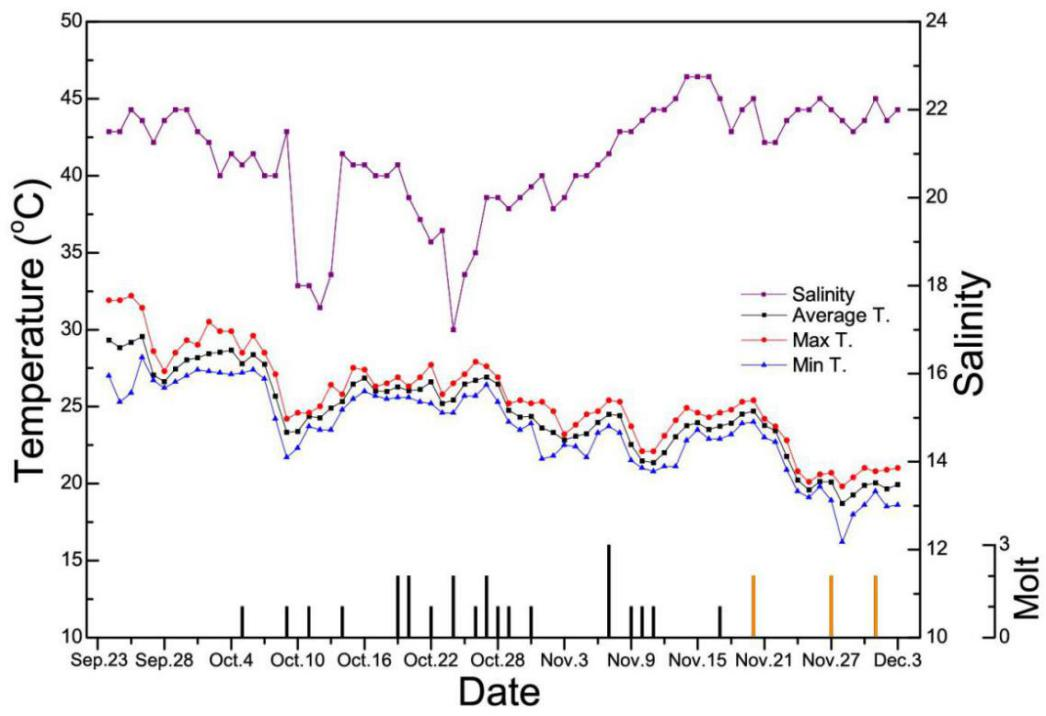

Supplement: FIG S4 [file msystems.00917-21-sf004.tif]

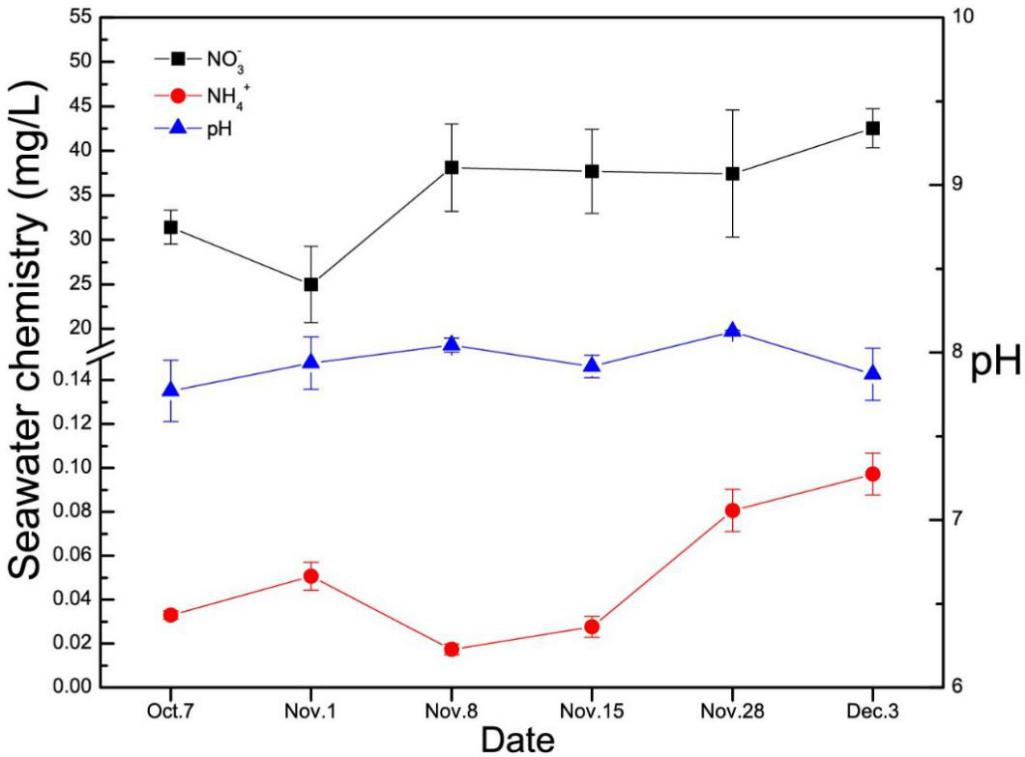

Supplement: FIG S5 [file msystems.00917-21-sf005.tif]
